# Thermal stability, pH dependence and inhibition of four murine kynurenine aminotransferases

**DOI:** 10.1186/1471-2091-11-19

**Published:** 2010-05-19

**Authors:** Qian Han, Tao Cai, Danilo A Tagle, Jianyong Li

**Affiliations:** 1 Department of Biochemistry, Virginia Tech, Blacksburg, Virginia 24061, USA; 2 OIIB, NIDCR, National Institutes of Health, Bethesda, Maryland 20892-4322, USA; 3 Neuroscience Center, NINDS, National Institutes of Health, Bethesda, Maryland 20892-9525, USA

## Abstract

**Background:**

Kynurenine aminotransferase (KAT) catalyzes the transamination of kynunrenine to kynurenic acid (KYNA). KYNA is a neuroactive compound and functions as an antagonist of alpha7-nicotinic acetylcholine receptors and is the only known endogenous antagonist of N-methyl-D-aspartate receptors. Four KAT enzymes, KAT I/glutamine transaminase K/cysteine conjugate beta-lyase 1, KAT II/aminoadipate aminotransferase, KAT III/cysteine conjugate beta-lyase 2, and KAT IV/glutamic-oxaloacetic transaminase 2/mitochondrial aspartate aminotransferase, have been reported in mammalian brains. Because of the substrate overlap of the four KAT enzymes, it is difficult to assay the specific activity of each KAT in animal brains.

**Results:**

This study concerns the functional expression and comparative characterization of KAT I, II, III, and IV from mice. At the applied test conditions, equimolar tryptophan with kynurenine significantly inhibited only mouse KAT I and IV, equimolar methionine inhibited only mouse KAT III and equimolar aspartate inhibited only mouse KAT IV. The activity of mouse KAT II was not significantly inhibited by any proteinogenic amino acids at equimolar concentrations. pH optima, temperature preferences of four KATs were also tested in this study. Midpoint temperatures of the protein melting, half life values at 65°C, and pKa values of mouse KAT I, II, III, and IV were 69.8, 65.9, 64.8 and 66.5°C; 69.7, 27.4, 3.9 and 6.5 min; pH 7.6, 5.7, 8.7 and 6.9, respectively.

**Conclusion:**

The characteristics reported here could be used to develop specific assay methods for each of the four murine KATs. These specific assays could be used to identify which KAT is affected in mouse models for research and to develop small molecule drugs for prevention and treatment of KAT-involved human diseases.

## Background

The aminotransferase capable of catalyzing the transamination of kynurenine to kynurenic acid (KYNA) using various co-substrates, has commonly been termed kynurenine aminotransferase (KAT). KYNA is the only known endogenous antagonist of the *N*-methyl-D-aspartate subtype of glutamate receptors[1-4]. It is also an antagonist of the α7-nicotinic acetylcholine receptor[5-8]. In addition, KYNA is identified as an endogenous ligand for an orphan G-protein-coupled receptor (GPR35) that is predominantly expressed in immune cells[9]. Abnormal concentration of KYNA in cerebrospinal fluid/brain tissue has been observed in patients with mental and neurological disorders, including the Huntington's disease, Alzheimer's disease, schizophrenia, multiple sclerosis and others (for a review see [10]). These data suggest that KYNA, acting as an endogenous modulator of glutamatergic and cholinergic neurotransmission, may be functionally significant in the development and progression of these diseases. In addition to its roles as an excitatory amino acid and α7-nicotinic acetylcholine antagonist, KYNA is also involved in the control of the cardiovascular function by acting at the rostral ventrolateral medulla of the central nervous system (CNS)[11]. Spontaneously hypertensive rat, the most widely used animal model for studying genetic hypertension, is associated with abnormally low KYNA levels in the area of CNS which controls physiological blood pressure[12,13].

KYNA is produced enzymatically by irreversible transamination of kynurenine, the key intermediate in the tryptophan catabolic pathway. In humans, rats and mice, four proteins arbitrarily named KAT I, II, III and IV, have been considered to be involved in KYNA synthesis in the CNS[14-20]. KAT I is identical to glutamine transaminase K (GTK) and cysteine conjugate beta-lyase (CCBL) 1; KAT II is identical to aminoadipate aminotransferase (AADAT); KAT III is identical to CCBL 2; and KAT IV is identical to glutamic-oxaloacetic transaminase (GOT) 2 and mitochondrial aspartate aminotransferase (ASAT). Although the involvement of these enzymes in brain KYNA production has been discussed, their specific roles in brain KYNA synthesis remain to be established.

Among the individual mammalian KATs, KAT I and KAT III share similar genomic structure and high sequence identity [18] and therefore likely have overlapped biological functions. An increase in KAT I and KAT III expression was observed in kat-2 ^-/- ^mice brain, suggesting that KAT I and KAT III expression compensated for the loss of KAT II [18]. This also might explain why phenotypes such as the hyperactivity and abnormal motor coordination in the kat-2 ^-/- ^mice were rescued[7,18,21]. These data suggest the importance of mammalian KAT I and KAT III in maintaining KYNA level in kat-2 ^-/- ^mouse brain.

There have been many studies dealing with the biochemical characteristics of mammalian KAT I and KAT II[15,17,22-26]. The crystal structures of human KAT I [27,28] and its homologues, glutamine-phenylpyruvate aminotransferase from *Thermus thermophilus *HB8 [29] and KAT from a mosquito, *Aedes aegypti *[30], have been determined. The crystal structure of human KAT II [26,31,32] and its homologues from *Pyrococcus horikoshii *[33] and *Thermus thermophilus *[34] have also been determined. The biochemical function and structural characteristics of mouse KAT (mKAT) III have been determined[20]; and there have been a number of studies concerning the biochemical characterization of KAT IV[19,35-38]. In this study, we functionally expressed mKAT I, II, III, and IV in the same expression system, purified their recombinant proteins, investigated their pH optima, temperature preferences, and identified specific inhibitors for each individual mKAT. These properties of mKATs I, II, III, and IV will help develop specific assays for the four enzymes, which could be used to identify which KAT is affected in mouse disease models."

## Results

### Biophysical properties of mKATs

**Figure 1:**
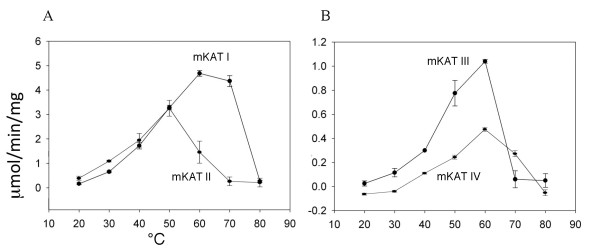
**Effect of temperature on enzyme activity**. The activities of recombinant mKATs at different temperatures. a) mKAT I and mKAT II, b) mKAT III and mKAT IV.

**Figure 2:**
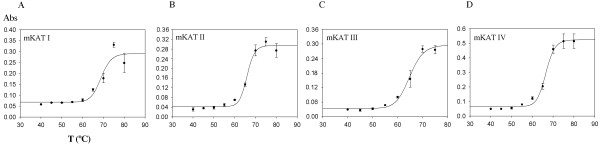
**The absorbance changes at 280 nm of four mKAT proteins after heat treatment**. a, mKAT I; b, mKAT II; c, mKAT III, and d, mKAT IV.

**Figure 3:**
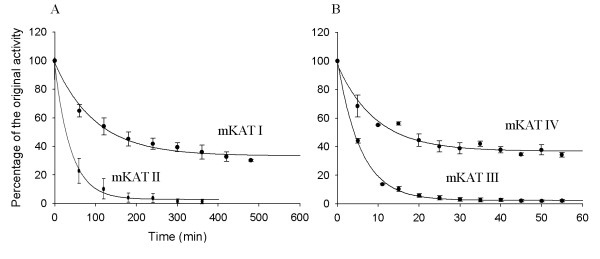
**Thermal stability of four mKAT proteins at 65°C**. a, Decay curves of mKAT I and mKAT II; b, mKAT III and mKAT IV.

SDS-PAGE analysis showed single band for each recombinant protein (not shown). Freezing and thawing did not alter the activity of the isolated enzymes. mKAT I showed maximum activity at 60 to 70°C (Fig. 1a), mKAT II showed maximum activity at 50°C (Fig. 1a), both mKAT III and mKAT IV (Fig. 1b) showed maximum activity at 60°C. The fact that all four enzymes showed high activity at 50°C, with mKAT I and IV functioning at 70°C, indicates they are resistant to heating. T_m _(midpoint temperature of the protein melting) values of mKAT I, II, III, and IV are 68.8 ± 1.2, 65.9 ± 0.5, 64.8 ± 0.7, 66.5 ± 0.5°C, respectively. Fig. 2 shows the fitting curve of each enzyme during the denaturation by heat treatment. The calculated half life in first order kinetics (t_1/2_) values of mKAT I, II, III, and IV at 65°C are 69.7 ± 8.8, 27.4 ± 3.2, 3.9 ± 0.1 and 6.5 ± 0.7 min, respectively (Fig. 3).

**Figure 4:**
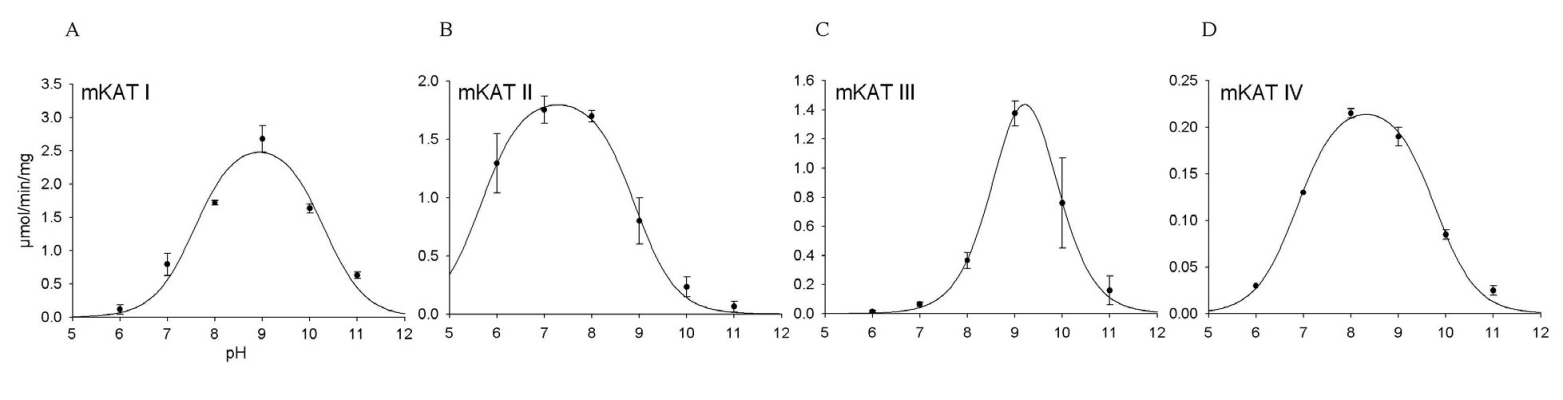
**Effect of pH on enzyme activity**. The activities of recombinant mKATs at different pH values. a) mKAT I, b) mKAT II, c) mKAT III, d) mKAT IV.

mKAT I, II, and IV all had a broad optimum pH range, while mKAT III had a relatively narrow optimum pH range. pH profiles of the four KAT proteins are all bell-shaped curves (Fig. 4). mKAT I and III showed maximum activity at pH 9 (Fig. 4a & 4c), mKAT II showed maximum activity at pH 7 to 8 (Fig. 4b), and mKAT IV (Fig. 4d) showed maximum activity at pH 8. The calculated pKa values of mKAT I, mKAT II, mKAT III, and mKAT IV are 7.6 ± 0.2, 5.7 ± 0.1, 8.7 ± 0.3 and 6.9 ± 0.1, respectively. The calculated pKb values of mKAT I, mKAT II, mKAT III, and mKAT IV are 10.3 ± 0.2, 5.7 ± 0.1, 8.7 ± 0.2 and 6.9 ± 0.1, respectively. Comparison of the pH optima of mKAT II and human KAT II, indicates that the pH optimum of mKAT II is apparently shifted toward more acidic conditions, as it showed higher activity at pH 6 than at pH 9, in contrast to human KAT II which showed much higher activity at pH 9 than at pH 6. The activity of mKAT II at pH 6 is not shared by the other three mKATs, which suggests a possible test for the specific activity of mKAT II. The pH optimum of mKAT I is similar to that of human KAT I [17], while the optimum of mKAT III expressed in *Escherichia coli *is the same as that of mKAT III expressed in insect cells[20].

### Substrate specificities of mKAT IV

**Figure 5:**
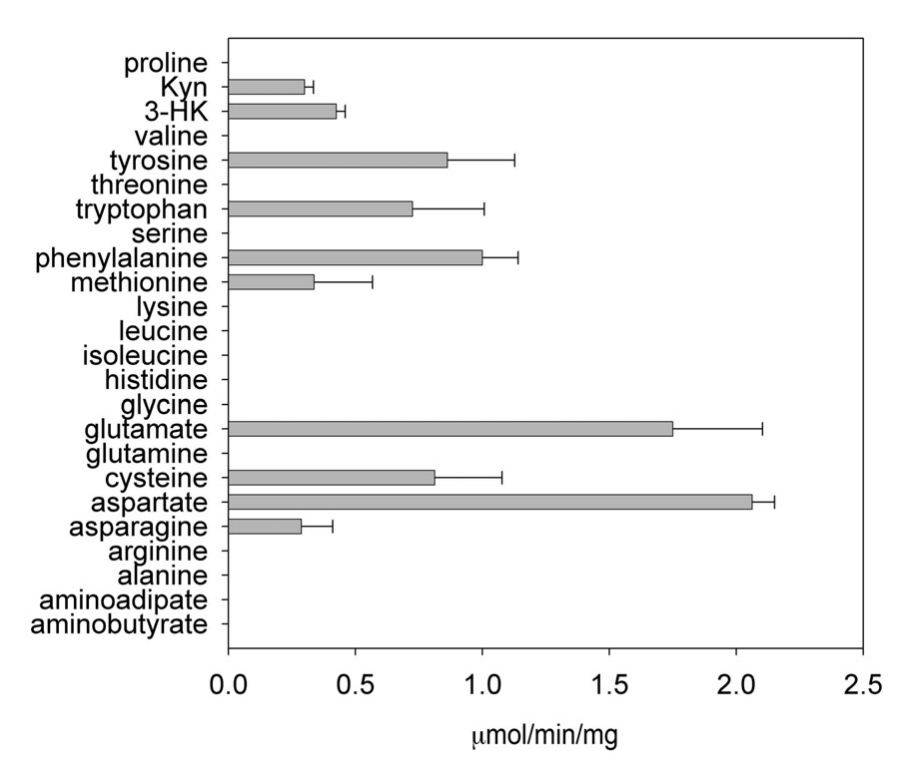
**Transamination activity of mKAT IV towards different amino acids with α-ketoglutarate or oxaloacetate as an amino group acceptor**. The activity was quantified by the amount of glutamate or aspartate produced in the reaction mixture. Oxaloacetate was only used for testing KAT activity with glutamate. 3-HK, 3-hydroxy-DL-kynurenine, Kyn: kynurenine.

mKAT IV was tested for aminotransferase activity towards 24 different amino acids using α-ketoglutarate (or oxaloacetate for glutamate), as a primary amino group acceptor. The enzyme showed the highest activity with aspartate and glutamate as substrates, but also showed activity with other amino acids, including some aromatic amino acids [phenylalanine, kynurenine, tryptophan, 3-hydroxy-DL-kynurenine (3-HK) and tyrosine] and sulfur containing amino acids (methionine and cysteine) (Fig. 5).

### Effects of other amino acids on four mKATs

**Figure 6:**
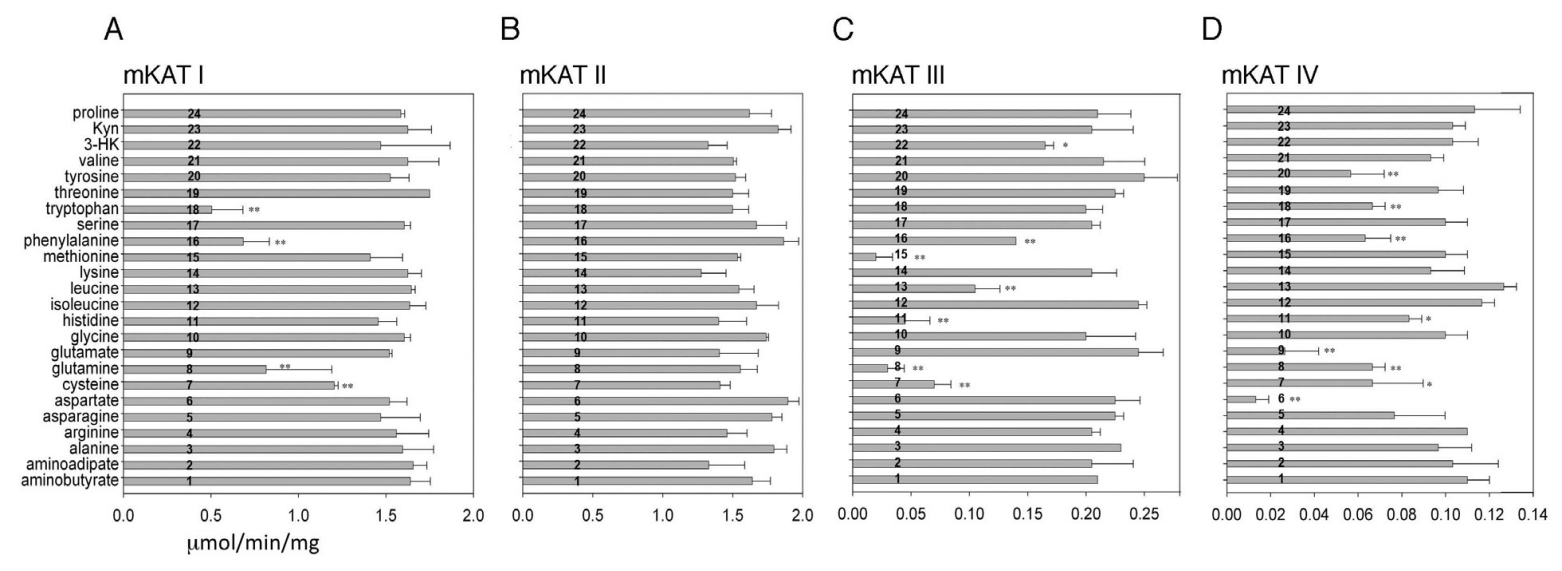
**Effect of other amino acids on mKAT enzyme activities**. The activities were quantified by the amount of KYNA produced in the reaction mixtures in 100 mM phosphate, pH 7.5. The mixture contains 5 mM kynurenine, 2 mM α-ketoglutarate (for mKAT I and III) or glyoxylate (for mKAT I and III), 5 mM other amino acid. The rate of KYNA production for each mKAT and different reaction mixture is shown in the figure. The bars labeled with stars (**P < 0.01, * P < 0.05) are significant different from the control (Kyn). Kyn: kynurenine, 3-HK, 3-hydroxy-DL-kynurenine. a) mKAT I, b) mKAT II, c) mKAT III, d) mKAT IV.

The inhibition of each of 23 other amino acids was investigated by incorporating them individually into reaction mixtures containing 5 mM kynurenine and each of the mKATs in turn. Of these, tryptophan, phenylalanine, glutamine, and cysteine were observed to inhibit mKAT I activity; methionine, glutamine, histidine, cysteine, leucine, and phenylalanine to inhibit mKAT III activity; and, aspartate and glutamate to inhibit around 80% and tyrosine, phenylalanine, tryptophan, glutamine, cysteine, asparagine, and histidine to inhibit 20 - 40% of mKAT IV activity. Although aminoadipate, lysine, 3-HK show inhibition of mKAT II activity, none of them inhibit more than 30% activity (Fig. 6).

### KAT activities of mouse brain crude proteins

**Figure 7:**
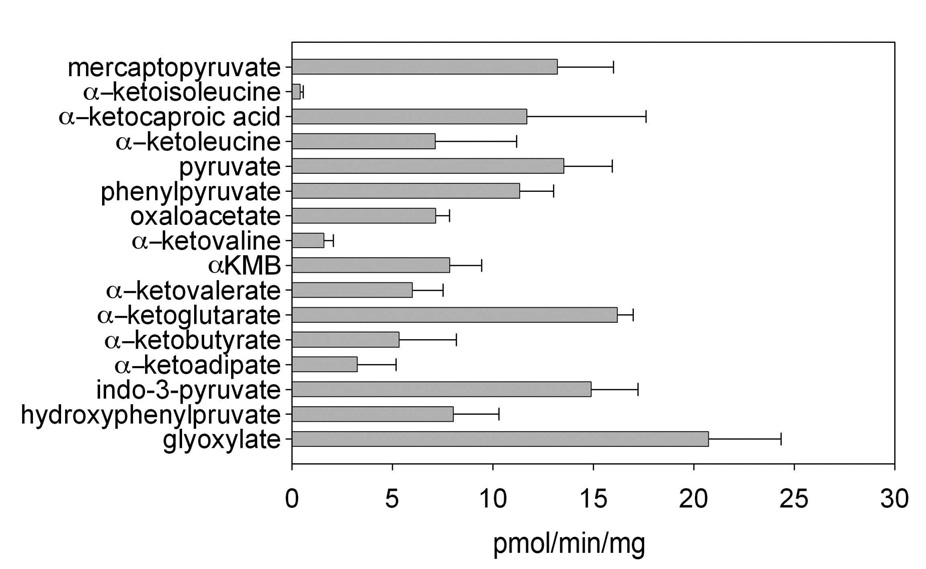
**Transamination activity of mouse brain crude protein extract towards different α-ketoacids**. The reaction mixture consists of 5 mM kynurenine, 2 mM α-keto acid, 40 μM PLP, 20 μg brain crude protein extract in 100 μL 100 mM phosphate buffer, pH 7.5. The mixture was incubated at 38°C for 2 h and the reaction was stopped by adding an equal volume of 0.8 M formic acid. Measurement of KYNA was performed by HPLC with fluorometric detection at Exc. 340 nm and Em. 398 nm.

The brain crude proteins showed KAT activity with all 16 tested α-ketoacids (Fig. 7). In particular, the crude proteins showed high activity with glyoxylate, a good co-substrate for mKAT I and III; and with α-ketoglutarate, a good substrate for mKAT II and IV. These results are consistent with previous studies which demonstrated that mouse brains have all four KAT enzymes[18-20].

**Figure 8:**
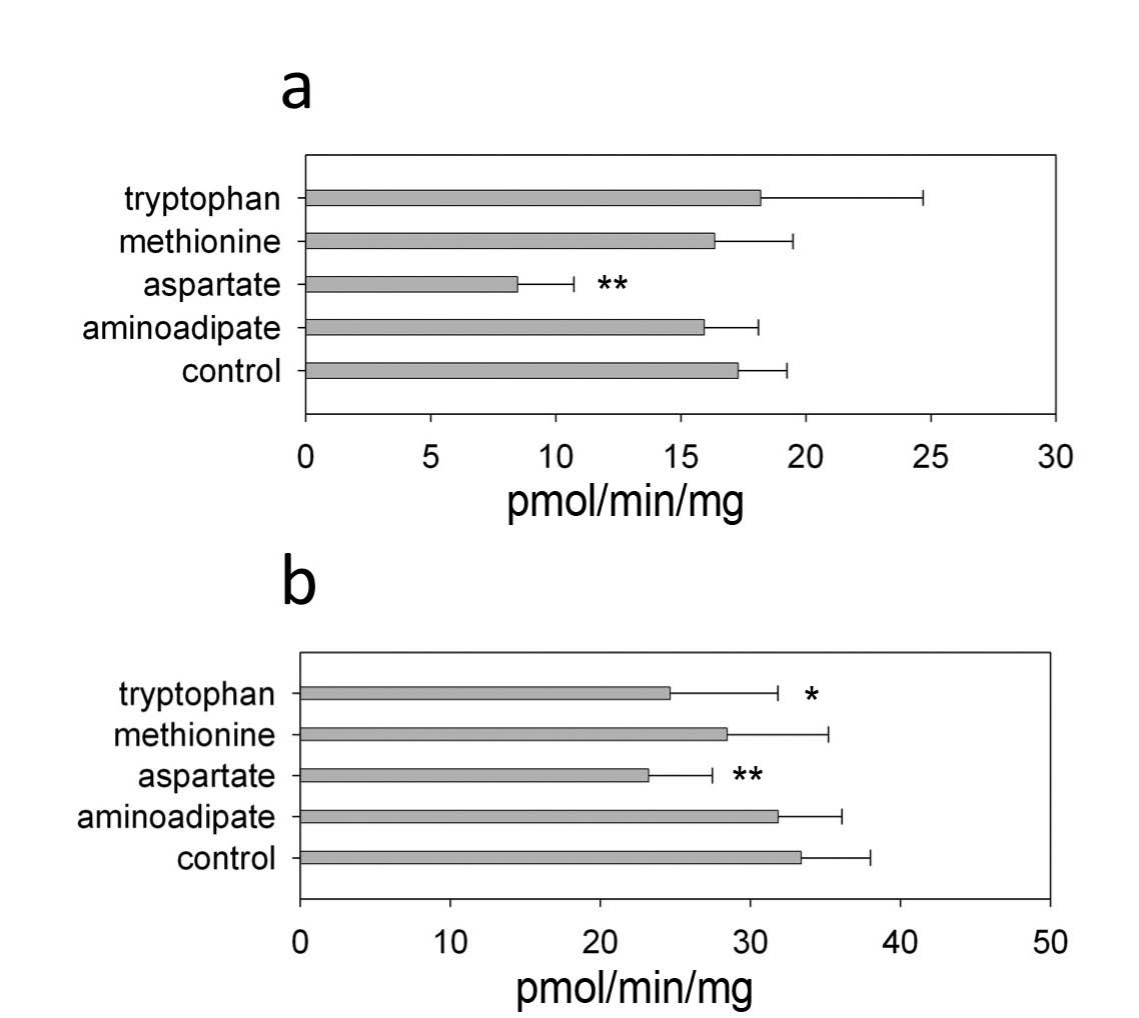
**Effect of four other amino acids on KAT activity of mouse brain crude protein extract**. The reaction mixture consists of 5 mM kynurenine, 2 mM co-substrate (glyoxylate or α-ketoglutarate), 40 μM PLP, 5 mM other amino acid (putative inhibitor), 20 μg brain crude protein extract in 100 μL 100 mM phosphate buffer, pH 7.5 or 100 mM sodium borate buffer, pH 9. The mixture was incubated at 38°C for 2 h. The reaction was stopped by adding an equal volume of 0.8 M formic acid. Measurement of KYNA was performed by HPLC with fluorometric detection at Exc. 340 nm and Em. 398 nm. a) Results at pH 7.5 using α-ketoglutarate as a co-substrate, b) Results at pH 9 using glyoxylate as a co-substrate.

Based on the aforementioned results of amino acid inhibition study, we used methionine, tryptophan, aminoadipate, and aspartate to test inhibition of KAT activity in mouse brain crude protein. We found that aspartate inhibited 48% KAT activity in the crude protein at pH 7.5 using α-ketoglutarate as a co-substrate (Fig. 8a) and 30% activity at pH 9 (Fig. 8b) using glyoxylate as a co-substrate. Tryptophan inhibited 26% and methionine inhibited 15% KAT activity at pH 9 using glyoxylate as a co-substrate (Fig. 8b). Aminoadipate did not significantly inhibit KAT activity at either pH 9 or pH 7.5.

## Discussion

The identity of ASAT with KAT was first reported in *E. coli *[39], and when later it was found that mitochondrial ASATs in mice, rats, and humans had KAT activity, it was also named KAT IV[19]. In this study, we showed mKAT IV has high transamination activity towards glutamate and aspartate, and detectable activity towards phenylalanine, tyrosine, cysteine, tryptophan, 3-HK, methionine, kynurenine, and asparagine. KAT IV was considered as a major player for the formation of KYNA in mouse, rat and human brains[19]. Our results certainly show that mouse brain protein extract has high mKAT IV activity as evidenced by the strong aspartate inhibition of the KYNA formation in the reaction mixture. High KAT activity is detected in mouse brain crude proteins using α-ketoglutarate as a co-substrate at neutral pH condition, and as mKAT IV is majorly responsible for this KAT activity, its contribution in brain KYNA synthesis has yet to be excluded. A gene knock-out study in animal models could address this question definitively.

KYNA is a metabolite in the kynurenine pathway, a major pathway in tryptophan metabolism, and its formation from tryptophan is a complicated process involving a number of enzymes [16,40]. The overall process leading to the formation of KYNA includes oxidation of tryptophan to formylkynurenine catalyzed by tryptophan 2,3 dioxygenase and indoleamine 2,3 dioxygenase; hydrolysis of formylkynurenine to kynurenine catalyzed by kynurenine formamidase; and transamination of kynurenine to KYNA catalyzed by four KATs. Alternatively, kynurenine could be oxidized by kynurenine 3-monooxygenase to form 3-HK or hydrolyzed by kynureninase to form anthranilic acid. The mouse brain crude extract not only consists of four KAT enzymes but also other proteins involved in the kynurenine pathway. In our preliminary studies, when we tested KAT activity at pH 7.5, we observed the formation of 3-HK and anthranilic acid [confirmed using standard compounds (not shown)]; we also noticed that KYNA formation was increased by adding tryptophan to the reaction mixture. This is perhaps because indoleamine 2,3 dioxygenase and tryptophan 2,3 dioxygenase oxidize tryptophan at the start of the kynurenine pathway, increasing the kynurenine concentration, which, in turn, results in increased KYNA formation. Since kynurenine 3-monooxygenase oxidizes kynurenine to 3-HK and kynureninase hydrolyzes kynurenine to anthranilic acid, they may compete with the mKATs for the same substrate, kynurenine, thus interfering with the KAT activity assay. The brain crude extract sample also contains amino acids and α-keto acids, which are substrates or inhibitors of four mKATs; NADH and NADPH, which are cofactors of kynurenine 3-monooxygenase; and hemin, which functions as a cofacor of both indoleamine 2,3 dioxygenase and tryptophan 2,3 dioxygenase. The presence of these substances and other kynurenine pathway enzymes could directly affect KAT activity assay by providing a co-substrate for KAT enzymes or by competitively inhibiting the enzymes; or they could indirectly change KAT activity by enhancing the activity of kynureninase or kynurenine 3-monooxygenase which competitively use kynurenine. To prevent or decrease the interference from other kynurenine pathway enzymes and brain chemicals, we first dialyzed the brain crude sample with a 10 kDa molecular weight cutoff membrane to eliminate possible substrates and inhibitors of four KAT enzymes, and cofactors of other kynurenine pathway enzymes from the sample. Herein, we demonstrated all mKAT I, III, IV showed high resistance to heat treatment, and mKAT II also showed noticeable activity at 60°C. The T_m _value of mKAT II is similar to that of other mKATs except that its activity declines at 60°C, which is lower than the T_m _value. One possible reason may be that the cofactor binding or dimer interaction is disrupted at 60°C, without changing the absorbance at 280 nm significantly. Spectral changes in 280 nm caused by protein denaturation are attributed to changes in the environment of tyrosyl and tryptophyl side chains, which are buried in the hydrophobic region of the native protein molecule [41]. So the contribution of this spectral change is made mainly by the disruption of secondary and tertiary structures. The damage of quaternary structure may not significantly contribute to this change unless there are enough tyrosine and tryptophan residues involved in dimerization. Comparing the crystal structures of the four mKATs will help elucidate the mechanism. CD spectroscopy of mouse indoleamine 2,3 dioxygenase indicated that the T_m _value is 60°C, with a second intermediate T_m _at 44°C[42], and that rabbit indoleamine 2,3 dioxygenase was not stable at 55°C[43]. Mosquito kynurenine 3-monooxygenase shows very little activity at 55°C[44], but there is little information about the thermal stability of mammalian kynurenine 3-monooxygenases. Here, we did notice that 3-HK formation is almost undetectable in the reaction mixture at pH 7.5 after dialysis and heat treatment of brain crude extract at 60°C for 15 min (not shown). Apparently, kynurenine 3-monooxygenase was deactivated either by taking away a cofactor or by destroying the secondary, tertiary or quaternary structure with heat treatment. Mammalian kynureninase has been studied for its substrate specificity, pH optimum and inhibition[45-47], but there is little information about its thermal stability. The heat treatment of the crude extract at 60°C for 15 min did decrease the anthranilic acid production catalyzed by kynureninase (data not shown). However, further study of the biochemical and biophysical properties of mouse kynureninase, indoleamine 2,3 dioxygenase, tryptophan 2,3 dioxygenase, and kynurenine 3-monooxygenase is needed in order to avoid the interferences with the KAT specific assays from these kynurenine pathway enzymes.

The purpose of comparing four different mKATs is to find their unique characteristics and to develop a sensitive and specific method to assay each mKAT. The assay previously reported for KAT I using pyruvate as a co-substrate at pH 9.5 [15] would also detect activity of the other three KATs, because they all can use pyruvate as an amino group acceptor and have activity at this pH[17,20]. In addition, pyruvate is not a favored co-substrate of KAT I. The commonly used KAT II assay [15] is also problematic because under the same assay conditions, KAT I and III have detectable activity and KAT IV has high activity[17,20]. mKAT enzymes might not have exactly the same properties as their human homologues, which have been characterized previously [17,26], we used the same protein expression system to produce all four mKATs and obtained pH profiles, temperature preferences and enzyme inhibitions for each individual enzyme. Some of the results are interesting, e.g. optimum pH of mKAT II (pH 6-8) is different from that of human KAT II (pH 7-9) [26]. The characteristics of each mKAT, which could be used for developing specific assays of four mKAT enzymes are summarized for clarity in table 1. Based on this information, we did preliminary tests to develop a specific assay of each KAT. The assays for mKAT I and mKAT II were shown to be highly specific; and the assay methods for mKAT III and IV were shown to be specific (Additional File 1).

**Table 1:** Major differences of four mKAT enzymes

	mKAT I	mKAT II	mKAT III	mKAT IV
T_m _(°C)	69.8	65.9	64.8	66.5

t_1/2 _(min)	69.7	27.4	3.9	6.5

pKa	7.6	5.7	8.7	6.9

pH optima	9	7-8	9	8

Specific co-substrate	α-ketobutyrate	α-ketoglutarate	α-ketobutyrate	α-ketoglutarate

Inhibitor	Indole-3-propionic acid		methionine	aspartate

T_m_: T*he midpoint *temperature *of the protein melting*; t_1/2_: half life value at 65°C

## Conclusions

This is the first kinetic characterization of the temperature and pH effects of the four mKAT enzymes. The characteristics reported here could be used to develop specific assay methods for each of the four murine KATs. These specific assays could be used to identify which KAT is affected in mouse models for research and to develop small molecule drugs for prevention and treatment of KAT-involved human diseases.

## Methods

### First-strand mouse brain cDNA synthesis and amplification of mKAT I, II, III and IV

Mouse brain was dissected from a euthanized male C57BL/6 mouse and rapidly frozen in liquid nitrogen. A mortar and a pestle, which were pre-cooled in liquid nitrogen, were used to pulverize the frozen mouse brain in the presence of additional liquid nitrogen. After the brain sample was pulverized to a fine powder, it was rapidly transferred into TRIZOL reagent (Gibco-BRL) for the isolation of total RNA. Isolated total RNA was used to synthesize first-stranded total brain cDNA. Oligonucleotide primers (Table 2) corresponding to individual mKATs were synthesized and used for amplification of their corresponding cDNA from the synthesized mouse brain cDNA pool.

**Table 2:** Oligonucleotide primers used for amplification of mKATs

Genes	Nucleotide Sequences	Restriction Sites
mKAT I (NP_765992)	5'-AAAACATATGTCCAAACAGCTGCAGGCTC-3' 5'-AAAACTCGAGTCAGGCTTGGGGCTCTCCTT-3'	Nde I Xho I

mKAT II (NP_035964)	5'-AAAACATATGAATTACTCACGGTTCCTCACT-3' 5'-AAAACTCGAGTCATAAAGACTCTTTTATCAGTTGGGC-3'	Nde I Xho I

mKAT III (AAQ15190)	5'-AAAAGAATGCTGCTTTGAAATTCAAAAACG-3' 5'-AAAAGAATTCTCAGTTCCAGGCCCTGAAGA-3'	Bsm I Eco RI

mKAT IV (NP_034455)	5'-AAAACATATGGCCAGAGCCAGCTCCT-3' 5'-AAAACTCGAGTTACTTGGTGACCTGGTGAATG-3'	Nde I Xho I

Note: The restriction sites are underlined.

### Expression and purification of recombinant mKATs

Each amplified sequence was cloned into an Impact™-CN plasmid (New England Biolabs) for expression of a fusion protein containing a chitin-binding domain. Transformed *E. coli *cells were cultured at 37°C. After induction with 0.2 mM IPTG, the cells were cultured at 15°C for 24 hrs. Two, six, three and three liters of cells were harvested as the start materials of mKAT I, II, III and IV, respectively, for affinity purification. The soluble fusion proteins were applied to a column packed with chitin beads and subsequently hydrolyzed under reducing conditions. The affinity purification resulted in the isolation of each individual KAT at around 80% purity. Further purifications of the recombinant KATs were achieved by DEAE-Sepharose, Mono-Q and gel-filtration chromatography. The purified recombinant KATs were concentrated to 10 mg ml^-1 ^protein in 10 mM phosphate buffer (pH 7.5) containing 40 μM PLP and 10 mM β-mercaptoethanol using a Centricon YM-50 concentrator (Millipore). Protein concentration was tested by a protein assay kit from Bio-Rad (Hercules, CA) using bovine serum albumin as a standard.

### KAT Activity assay

The KAT activity assay was based on previously described methods[17,20,26]. Briefly, a reaction mixture of 100 μL, containing 5 mM L-kynurenine, 2 mM α-ketoacids (α-ketoglutarate for mKAT II and IV, glyoxylate for mKAT I and III), 40 μM PLP, and 5 μg of recombinant protein, was prepared using 100 mM potassium phosphate buffer (pH 7.5). This reaction mixture will be identified hereafter as the typical mixture. The mixture was incubated for 15 min at 38°C, and the reaction was stopped by adding an equal volume of 0.8 M formic acid. The supernatant of the reaction mixture, obtained by centrifugation at 15,000 ***g ***for 10 min at room temperature, was analyzed for the product, KYNA, by high-performance liquid chromatography (HPLC) with ultraviolet detection at 330 nm.

### Substrate specificity of mKAT IV

The transamination activity of mKAT IV to other amino acids was tested using an assay mixture containing 10 mM of an amino acid, 10 mM α-ketoglutarate (or 10 mM oxaloacetate for the activity assay of glutamate), 40 μM PLP, 100 mM phosphate buffer, pH 7.5, and 5 μg enzyme in a total volume of 100 μl. The mixture was incubated for 15 min at 38°C and stopped by adding an equal amount of absolute ethanol. The product was quantitated based on the detection of o-phthaldialdehyde thiol (OPT)-amino acid product conjugate by HPLC with electrochemical detection after their corresponding reaction mixtures were derivatized by OPT reagent[39].

### Effect of pH and temperature on recombinant mKAT enzymes

To determine the effect of buffer pH on the activity of the four mKATs, a buffer mixture consisting of 100 mM phosphate and 100 mM boric acid was prepared and the pH of the buffer was adjusted to 6.0, 7.0, 8.0, 9.0, 10.0 and 11.0. The substrates and PLP were used in the same concentrations as described above for the specific activity assay. pKa and pKb values of two proteins were calculated based on the data of specific activities versus pH values using SigmaPlot Enzyme Kinetics Module (SPSS, San Jose, CA). To determine the effect of temperature on the four mKAT enzymes, the typical reaction mixture in 100 μL of phosphate buffer (pH 7.5) was incubated at temperatures ranging from 20 - 80°C for 15 min. During the mixture preparations, enzymes were held at same temperature for 5 min.

In T_m _value tests, enzymes were diluted in 100 mM potassium phosphate buffer, pH 7.5. Each mKAT was then heated at various temperatures for 5 min using a thermal cycler (MJ Research, Waltham, MA). The absorbance at 280 nm was detected using a UV-Visible spectrophotometer (Hitachi, Tokyo, Japan). The T_m _value for each enzyme was calculated by fitting the equation to the recorded data using the standard curve analysis function in SigmaPlot Enzyme Kinetics Module (SPSS, San Jose, CA).

The thermal stability was determined by studying thermal inactivation of enzyme incubated in 50 mM phosphate buffer, pH 7.5, at 65°C using a thermal cycler (MJ Research, Waltham, MA). Periodically, samples of the incubated enzymes were removed, chilled immediately by mixing with a cold phosphate buffer, 50 mM, pH 7.5. The remaining activity was assayed within 10 min using same method described above. Rate constant (k) was calculated based on the equation

where e is a mathematical constant, approximately equal to 2.718281828, t is time (min), [A] is mole of the active enzyme left, [A_0_] is mole of the original protein. t_1/2 _is calculated based on the equation

where k is the rate constant (min^-1^), and ln is the natural logarithm.

### Effect of other amino acids on four mKAT activities

To determine the effect of other amino acids on mKAT enzymes, each amino acid was incorporated into 100 μL of the typical reaction mixture (5 mM kynurenine, 2 mM co-substrate, 100 mM phosphate buffer at pH 7.5) at a final concentration of 5 mM and the enzyme activity was assayed in the same manner as described for the KAT activity assay.

### KAT activity assay for mouse brain crude proteins

Five to seven week old BALB/c mice were used. All experimental procedures were approved by the Institutional Animal Care and Use Committee of Virginia Polytechnic Institute and State University and met or exceeded requirements of the Public Health Service/National Institutes of Health and the Animal Welfare Act. The animals were housed in groups of four to five per cage, in a room with controlled light/dark cycle (12 h light/12 h dark), and were given free access to laboratory chow and tap water. Two females and two males were sacrificed; the brains were immediately removed and frozen in liquid nitrogen. The brains were ground in liquid nitrogen, and the powder was transferred into 10 ml of 5 mM Tris-acetate buffer containing 40 μM PLP and 10 mM β-mercaptoethanol (extract buffer) at pH 8.0, and mixed well. The mixture was centrifuged at 20,000 g, 4°C, for 20 min. The supernatant was collected and dialyzed overnight at 4°C against the protein extract buffer with a 10 kDa molecular weight cutoff membrane. The dialyzed brain crude protein extract was used for KAT activity assay. The crude protein concentration was tested by a protein assay kit from Bio-Rad (Hercules, CA) using bovine serum albumin as a standard. We used 20 μg brain crude protein in 100 μl of the same typical reaction mixture as was used in the recombinant protein KAT activity assay. The mixture was incubated at 38°C for 2 h. Since the amount of KYNA formed by the brain crude extract in reaction mixture is quite low compared to that formed by the recombinant protein, measurement of KYNA was performed by HPLC with fluorometric detection according to the method described by Baran and Kepplinger[48]. The fluorescence detector was set at an excitation wavelength of 340 nm and an emission wavelength of 398 nm. The substrate specificity for α-keto acids and the inhibition of different amino acids of the brain crude extract were also tested. The inhibition was tested at both pH 7.5 (100 mM phosphate buffer) and pH 9 (100 mM sodium borate buffer).

## Abbreviations

3-HK: 3-hydroxy-DL-kynurenine; AADAT: aminoadipate aminotransferase; ASAT: aspartate aminotransferase; CCBL: cysteine conjugate beta-lyase; GTK: glutamine transaminase K; GOT: glutamic-oxaloacetic transaminase; KAT: kynurenine aminotransferase; mKAT: mouse kynurenine aminotransferase; KYNA: kynurenic acid; PLP: pyridoxal-5'-phosphate.

## Authors' contributions

QH participated in the design of the study, carried out the experiment, performed analysis and wrote the manuscript. TC participated in the design of the study and helped to draft the manuscript. DAT participated in the design of the study and helped to draft the manuscript. JL participated in the design of the study, carried out the experiment, and wrote the manuscript. All authors read and approved the final manuscript.

## Supplementary Material

Additional file 1 **Supplementary data**. The file shows the tests of specific activity assays for four mKAT enzymes. It contains the method, figure and figure legend. Click here for file
