# Supplementary material for: Thermal stability, pH dependence and inhibition of four murine kynurenine aminotransferases
Source: BMC Biochem. 2010 May 19;11:19. doi: 10.1186/1471-2091-11-19 (PMC2890522; doi:10.1186/1471-2091-11-19)
Supplement: Additional file 1 — Supplementary data. The file shows the tests of specific activity assays for four mKAT enzymes. It contains the method, figure and figure legend. [file 1471-2091-11-19-S1.PDF]

Supplemental data

Tests of specific activity assays for four mKAT enzymes.

Based on their available characteristics, potential specific activity assays were designed for each of the four mKAT enzymes. Each assay was then tested for specificity using all four enzymes.

Specific KAT activity assays for mKAT I: For the first assay, a 1 μg enzyme sample of the mKAT to be tested was heated to 70 ºC for 5 minutes, then added to a reaction mixture containing kynurenine (1 mM), α-ketobutyrate (1 mM), pyridoxal-5′-phosphate (PLP) (40 μM), and 100 mM boric acid at a final pH of 9. After incubation at 38 ºC for 15 minutes, the reaction was stopped by adding an equal volume of 0.8 M formic acid. The mixture was centrifuged at 15,000 g for 10 min and supernatant was analyzed by reverse-phase HPLC with fluorometric detection at Exc. 340 nm and Em. 398 nm for both kynurenine and KYNA. The activity of mKAT I was arbitrarily assigned to 100%, and the activities of the other mKATs were expressed as a percentage of mKAT I. The results are illustrated in Figure S1-a1. The second activity assay used the same preheat treatment and reaction mixture, but the incubation temperature was 70 ºC. The results are shown in Figure S1-a2. The third assay used the exact same method as the first, but with the addition of 1 mM aspartate. The results are shown in Figure S1-a3.

Specific KAT activity assays for mKAT II: For the first assay, a 1 μg enzyme sample of the mKAT to be tested was heated to 50 ºC for 5 minutes, then added to a reaction mixture containing kynurenine (1 mM), α-ketoglutarate (1 mM), PLP (40 μM), and 100 mM phosphate buffer, pH 6.0. After incubation at 38 ºC for 15 minutes, the same procedure as described for mKAT I was used to quantitate KYNA. The results are shown in Figure S1-b1. The second activity assay used the same preheat treatment and reaction mixture preparation, but with addition of 1 mM aspartate and 1 mM indole-3-propionic acid that previously showed strong inhibition to human KAT I. The results are shown in Figure S1-b2.

Specific KAT activity assay for mKAT III: For the first assay, a 1μg enzyme sample of the mKAT to be tested was heated to 60 ºC for 5 minutes, then added to a reaction mixture containing kynurenine (1 mM), α-ketobutyrate (1 mM), PLP (40 μM), and 100 mM boric acid at a final pH of 9.0. After incubation at 38 ºC for 15 minutes, the same procedure as described for mKAT I was used to quantitate KYNA. The result is shown in Figure S1-c1. The second activity assay used the same preheat treatment and assay procedures, but the incubation temperature was 60 ºC. The results are shown in Figure S1-c2. The third assay used the exact same method same as the first, but with the addition of 2 mM aspartate and 2 mM indole-3-propionic acid. The results are shown in Figure S1-c3.

Specific KAT activity assay for mKAT IV: For the first assay, a 1 μg enzyme sample of the mKAT to be tested was heated to 70 ºC for 5 minutes, then added to a reaction mixture containing kynurenine (1 mM), α-ketoglutarate (1 mM), PLP (40 μM), and 100 mM Tris buffer, pH 8. After incubation at 38 ºC for 15 minutes, the same method as described for mKAT I was used to detect KYNA. The result is shown in Figure S1-d1. The second activity assay used the same preheat treatment and reaction mixture preparation, but the incubation temperature was 70 ºC. The result is shown in Figure S1-b2. The third assay used the exact same method same as the second, but with addition of 2 mM indole-3-propionic acid. The result is shown in Figure S1-d3.

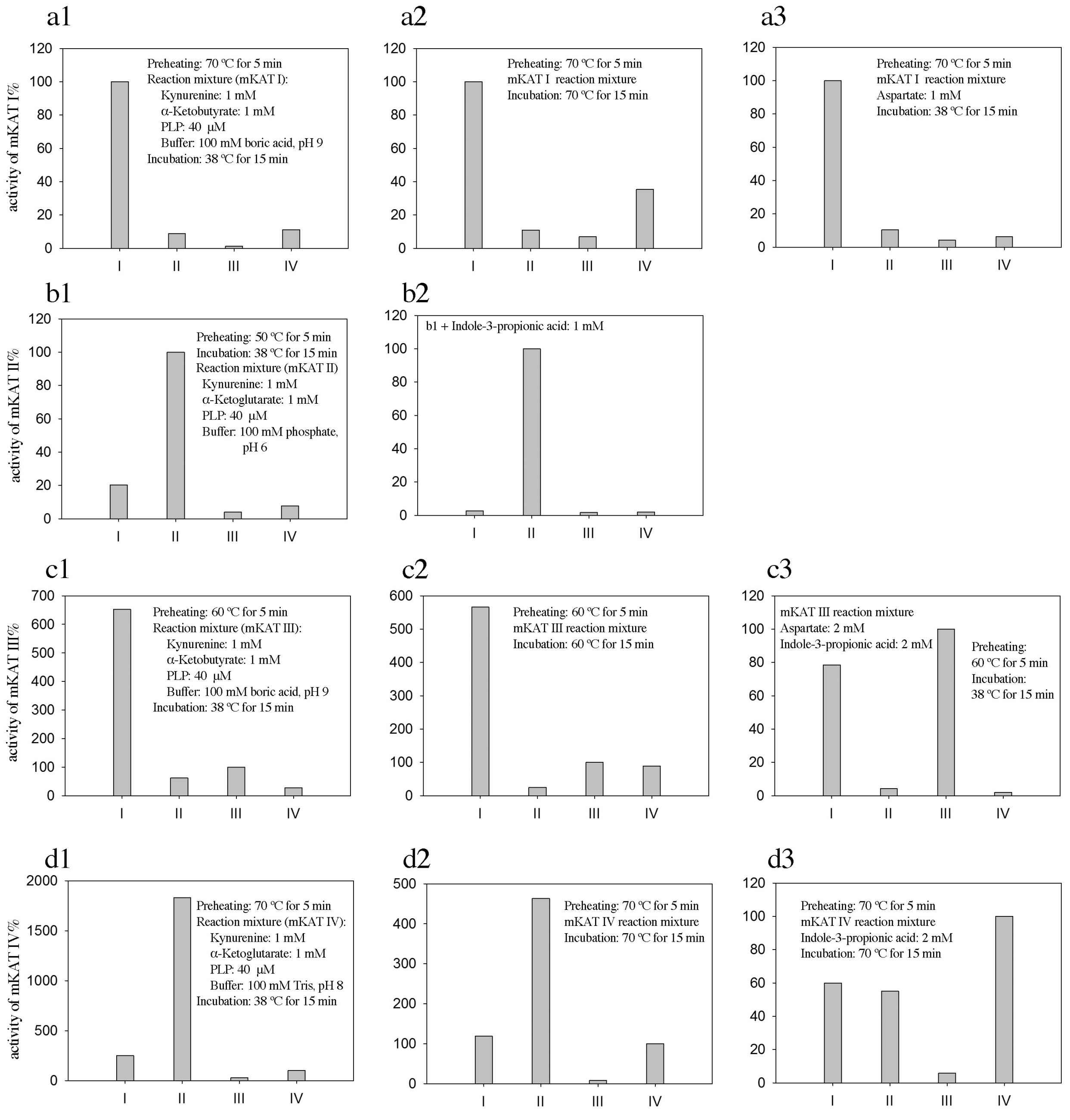

Figure S1. Tests of specific activity assay for each mKAT. The specific activities of mKAT I, mKAT II, mKAT III and mKAT IV are arbitrarily expressed as 100% in a(1-3), b(1-2), c(1-3) and d(1-3), respectively. Specific activities of the others are expressed as a percentage of the activities of mKAT I, mKAT II, mKAT III and mKAT IV in a1-3, b1-2, c1-3 and d1-3, respectively. a1) preheating: 70 ºC for 5 minutes, kynurenine: 1 mM, α-ketobutyrate: 1 mM, PLP: 40 μM, buffer: 100 mM boric acid, pH 9, incubation: 38 ºC for 15 minutes; a2) same as a1 but with different incubation temperature, 70 ºC; a3) same as a1 but with the addition of 1 mM aspartate; b1) preheating: 50 ºC for 5 minutes, kynurenine: 1 mM, α-ketoglutarate: 1 mM, PLP: 40 μM, buffer: 100 mM phosphate, pH 6, incubation: 38 ºC for 15 minutes; b2, same as b1 but with the addition of 1 mM aspartate and 1 mM indole-3-propionic acid and without preheating; c1, preheating: 60 ºC for 5 minutes, kynurenine: 1 mM, α-ketobutyrate: 1 mM, PLP: 40 μM, buffer: 100 mM boric acid, pH 9, incubation: 38 ºC for 15 minutes; c2, same as c1 but with different incubation temperature, 60 ºC; c3, same as c1 but with the addition of 2 mM aspartate and 2 mM indole-3-propionic acid, and without preheating; d1, preheating: 70 ºC for 5 minutes, kynurenine: 1 mM, α-ketoglutarate: 1 mM, PLP: 40 μM, buffer: 100 mM Tris, pH 8, incubation: 38 ºC for 15 minutes; d2, same as d1 but with different incubation temperature, 70 ºC; d3, same as d2 but with the addition of 2 mM indole-3-propionic acid. I, II, III, and IV represent mKAT I, mKAT II, mKAT III and mKAT IV, respectively.
